# Acetylated Nanocelluloses Reinforced Shape Memory Epoxy with Enhanced Mechanical Properties and Outstanding Shape Memory Effect

**DOI:** 10.3390/nano12234129

**Published:** 2022-11-22

**Authors:** Tianyu Yu, Feilong Zhu, Xiongqi Peng, Zixuan Chen

**Affiliations:** 1 School of Materials Science and Engineering, Shanghai Jiao Tong University, Shanghai 200030, China; 2 School of Mechanical Engineering, University of Shanghai for Science and Technology, Shanghai 200093, China

**Keywords:** nanocellulose, nanocomposite, shape memory epoxy, acetylation, thermomechanical property

## Abstract

Shape memory polymers (SMPs) have aroused much attention owing to their large deformation and programmability features. Nevertheless, the unsatisfactory toughness and brittleness of SMPs still restrict their practical intelligent applications, e.g., textiles, flexible electronics, and metamaterials. This study employed nature-derived nanocelluloses (NCs) as the reinforcement to fabricate shape memory epoxy-based nanocomposites (SMEPNs). An acetylation modification approach was further proposed to ameliorate the intrinsic incompatibility between NCs and epoxy matrix. The storage modulus increases, and the shape memory effect (SME) sustains after acetylated nanocelluloses (ANCs) incorporation. The SMEPNs with 0.06 wt.% ANCs loading perform the most exceptional toughness improvement over 42%, along with the enhanced fracture strain, elastic modulus, and ultimate strength. The incorporated nanoscale ANCs effectively impede crack propagation without deterioration of the macromolecular movability, resulting in excellent mechanical properties and SME.

## 1. Introduction

Shape memory polymers (SMPs) are a class of innovative stimuli-responsive polymers that sustain shapes in stable environments and perform reversible deformation under external stimuli, e.g., temperature changes, illuminations, solvents, and magnetic fields [1,2]. Due to the advantages of excellent processability, low cost, large deformation, programmable thermal properties, and good biocompatibility, SMPs have demonstrated wide application prospects in recent years, for instance, space deployable structures, biomedical equipment, intelligent textiles, self-healing composite systems, optical reflectors, deformable skin structures, and automobile sensors [3,4,5,6,7]. In accordance with different crosslinking mechanisms, SMPs can be categorized to shape memory plastics with physically induced crosslinks (SMTPs) and thermosetting polymers with covalent crosslinks (SMTSs) as long as their molecular structure contains no less than two separate phases [8]. Compared with SMTPs, e.g., polyurethane, polynorbornene, crosslinked polyethylene, styrene rubber, and acrylate, SMTSs exhibit preferable thermal stability and stiffness [9,10]. Among the SMTSs family, shape memory epoxy polymers (SMEPs) are widely accepted for use in diverse areas due to their promising thermophysical property and high processability. However, the unsatisfactory toughness and brittleness of SMEPs restrict their applications in the most state-of-the-art high-end fields. To comprehensively enhance the properties of SMEPs, researchers are devoted to developing SMEPs-based composites by incorporating reinforcements like conventional fibers and nano-additives.

The size of nano additives is usually in the range of 1–100 nm, which is advantageous in bringing unique optical, electronic, thermal, and mechanical properties. Incorporating nano additives to produce shape memory polymeric nanocomposites (SMPNs) has been proven an effective method to modify the comprehensive properties of SMPs in mechanical, thermal, and electrical aspects [11]. Liu et al. [12] modified pristine shape memory epoxy by adding nano-scale SiC additives. The elastic modulus and recovery forces were significantly enhanced with 20 wt.% nano-additives loading. Liu et al. [13] found that the incorporation of organic montmorillonites in SMEPs improved the toughness, tensile strength, and shape recovery speed without deterioration of shape recovery ratio. Lu et al. [14] incorporated hybrid carbon nanofibers and sub-micro filamentary nickel nanostrands to SMEPs. The fabricated SMEPNs turned from insulative to conductive and can be activated by electrical resistive heating with accelerated response speed. Ding et al. [15] found that the glass transition temperature ($T_{g}$) and shape memory performance of silanized vapor-grown carbon nanofiber incorporated SMEPs were remarkably enhanced. Other studies also concluded that incorporating nanosized halloysite nanotubes, kaolinites, and micro-sized diatomites to basalt fiber reinforced epoxies is advantageous in improving the stiffness and interlaminar fracture toughness of SMEPs [16,17,18,19].

Nanocelluloses (NCs) are a type of natural nanomaterial that can be obtained from bacteria/microorganisms (xylose yeast), wood (cork, hardwood, recycled newspaper, and magazine fiber), non-wood lignocellulose (sisal, coconut, hemp, flax, jute, ramie, kenaf, cotton, and algae), and agricultural residues/by-products (corn cob, bagasse, banana, and crop straw) [20,21]. The preparation methods of NCs mainly include acid hydrolysis, enzymatic hydrolysis, electrospinning, oxidation, steam explosion, and ionic liquid, among which acid hydrolysis is the earliest and most comprehensive method in NCs preparation [22,23]. NCs have features of large aspect-ratio, large specific surface area, and high stiffness and strength, rendering them a promising candidate as the reinforcement for SMEPs [24]. Moreover, the nature-derived biodegradable NCs are the ideal alternatives to petroleum-based reinforcement materials like carbon fibers, glass fibers, carbon nanotubes (CNTs), etc. Auad et al. [25] prepared SMPNs by casting stable NCs/segmented polyurethane suspensions. Compared to neat polyurethane, the melting heat, elastic modulus, and tensile strength of the prepared SMPNs increased without obvious compromise on the recovery ratio. Song et al. [26] developed novel water-induced SMPNs by using hybrid reinforcements comprising graphene oxide (GO) and microcrystalline cellulose nanofibers (MSF-g-COOH) extracted from sisal fibers. The strong hydrogen bonding interaction between MSF-g-COOH and GO is favorable to enhance the elastic modulus and tensile strength by 0.5 wt.% GO loading. Gupta and Mekonnen [27] synthesized nanocrystalline celluloses (CNCs) enabled polycaprolactone-based SMPNs using an in situ one-pot reactions. Incorporation of up to 10 wt.% CNCs significantly improved yield strength, tensile strength, and elastic modulus while maintaining the elongation at break. Nevertheless, the highly polarized hydroxyl groups contribute to the hydrophilic nature of NCs, leading to inferior compatibility with hydrophobic and non-polar polymers. Moreover, driven by its high affinity, incompatible NCs can easily form self-assembled aggregates through strong hydrogen bonds, resulting in poor dispersion in the polymer matrix [28]. The defective interfacial adhesion and agglomeration of NCs are serious issues that restrict the performance of SMPNs. Therefore, physical or chemical surface modification of NCs is crucial to achieving a higher reinforcing effect. Up to date, diverse modification approaches are developed, including non-covalent modification [29], acylation [30], TEMPO-mediated oxidation [31], silanization [32], and grafting-onto/grafting-from methods [33]. Nevertheless, the comprehensive properties of SMEPNs reinforced by acylated ANCs are not yet been elucidated.

The objective of this study is to ameliorate the inherent incompatibility between nature-derived NCs and hydrophobic non-polar SMEPs by proposing an innovative acylation modification approach. By implementing acylation approach the hydroxyl groups among NCs are expected to be substituted with acetyl groups, thereby improving the hydrophobicity and compatibility with SMEPs. The feasibility of acylation is validated by composition analysis and dispersity test. The reinforcing effect by ANCs loading is comprehensively investigated regarding the mechanical properties and SME. Eventually, the optimal epoxy monomer to curing agent mole ratio and ANCs loading are explored, and the reinforcing mechanism by ANCs is elaborated.

## 2. Experiments

### 2.1. Materials

NCs (99.6%) were supplied by ScienceK Co., Ltd., Huzhou, China. Solvents including sulfuric acid (95.0–98.0%), acetic acid (≥99%), acetic anhydride (≥99%), acetone (≥99.5%), and ethanol (≥99.45%) for acetylation modification were all supplied by Aladdin Biochemical Technology Co., Ltd., Shanghai, China. Thermosetting Bisphenol A epoxy resin EPON 826 (weight per epoxide: 178–186 g/eq) was purchased from Hexion Co., Ltd., Columbus, OH, USA. Polyetheramine D230 (average Mn ~230%) was supplied by Aladdin Biochemical Technology Co., Ltd., Shanghai, China.

### 2.2. Acetylation Modification on NCs

Firstly, 1 g as-received NCs was mixed with 20 mL acetic acid, stewing for 1 h, and ultrasonication (TL-250Y, Jiangsu Tianling Instrument Co., Ltd., Yancheng, China) for 10 min for uniform dispersion. Then, 4 mL acetic anhydride and 0.05 mL concentrated sulfuric acid were added, and the suspension temperature was set to 50 °C in a thermostatic water bath (HH-1, Lichen Keyi Co., Ltd., Shanghai, China) for 1 h reaction. In this step, hydrogen in hydroxyl groups was gradually replaced by acetyl groups to realize the acetylation modification of NCs. Afterward, the suspension was cooled to room temperature, and ethanol was added to terminate the reaction. The white flocculent precipitates were collected and rinsed with distilled water to remove residual solvents. Finally, the synthesized acetylated nanocelluloses (ANCs) were dried in a vacuum oven (DZF-6020, Shanghai Jing Hong Laboratory Instrument Co., Ltd., Shanghai, China) at 60 °C for 12 h. During acetylation modification, acetic acid, acetic anhydride, and sulfuric acid served as the solvent, the acetylation agent, and the catalyzer, respectively.

### 2.3. Preparation of SMEPs and SMEPNs

To prepare SMEPs, 50 g epoxy monomers were firstly deaerated at 65 °C for 30 min. Specific amounts of curing agent were then added with 5 min magnetic stirring (MS6-Pro, DLAB Scientific Co., Ltd., Beijing, China), followed by another deaeration process at 65 °C for 10 min. The mixture was poured into a rectangular-shaped mold and cured at 100 °C for 1.5 h and 130 °C for 1 h. After curing, samples were cooled to room temperature at a relatively low cooling rate (approx. 1.5 °C/min) to minimize the effect of residual thermal and mechanical stresses. The thickness of fabricated samples was controlled to 2.0 mm. The detailed compositions of SMEPs are listed in Table 1.

SMEPNs were prepared by incorporating different amounts of ANCs into EP4. During preparation, specific amounts of the synthesized ANCs were dispersed in acetone with 30 min ultrasonication, 50 g epoxy monomers were then added with 30 min ultrasonication in cold water bath environment. Next, the mixtures were magnetic stirred for 5 h and heated at 65 °C for 12 h in a vacuum oven. After the complete removal of acetone, 18.05 g curing agent was added with 5 min magnetic stirring and 10 min deaeration at 65 °C. The prepared mixtures were then poured into a mold and performed an identical curing process with SMEPs. The detailed incorporation amounts of ANCs are listed in Table 2.

### 2.4. Characterizations

The constitutions and properties of the synthesized ANCs were comprehensively evaluated by transmission electron microscopy (TEM, Talos L120C G2, Thermo Fisher Scientific Inc., Waltham, MA, USA), Fourier-transform infrared spectroscopy (FTIR, Nicolet 6700, Thermo Fisher Scientific Inc., Waltham, MA, USA), X-ray diffraction (XRD, D8 ADVANCE Da Vinci, Bruker Co., Ltd., Billerica, MA, USA), and nuclear magnetic resonance (NMR, AVANCE NEO, Bruker Co., Ltd., Billerica, MA, USA).

Dynamic mechanical analysis (DMA, Q850, TA Instruments, New Castle, DE, USA) was conducted based on tension mode, and the geometry of specimens was 25 × 8 × 2 mm. The heating rate, frequency, and pre-load were set to 3 °C/min, 1 Hz, and 0.001 N, respectively. Tensile test was performed on a universal test machine with 1 mm/min test speed. Scanning electron microscopy (SEM, SIRION200, FEI Company, Hillsboro, OR, USA) was conducted on the fracture surfaces of SMEPNs.

The SME of prepared SMEPs and SMPENs was firstly determined by dynamic mechanical analysis approach under single cantilever beam mode with the following process: (i) dwell for 5 min at 20 °C above $T_{g}$, (ii) apply loading to 1.0 MPa with 0.1 MPa/min loading rate, (iii) dwell for 5 min, (iv) cooling to 20 °C with 5 °C/min cooling rate, (v) remove loading, dwell for 5 min at 20 °C, and record the fixed strain (vi) reheat to 20 °C above $T_{g}$ at 5 °C/min heating rate, record the residual strain after shape recovery.

To further evaluate the SME, a lab-made mold was employed to carry out the deployment test on 105 × 10 × 2 mm rectangular specimens following the sequences: (i) preheating the oven to 20 °C above $T_{g}$, (ii) transferring the specimen to the preheated oven, dwelling for 5 min, (iii) folding the specimens to 180° and fixing the shape, (iv) cooling at 10 °C in a refrigerator for 10 min, removing the mold, and leaving at room temperature for 30 min, (v) recording the shape change, and moving to the preheated oven to recovery. It is worth mentioning that shape changes during fold-unfold cycles were recorded by videos, whereby the angle at different time intervals can be conveniently measured.

## 3. Results and Discussions

### 3.1. Constituents and Properties of ANCs

**Figure 1:**
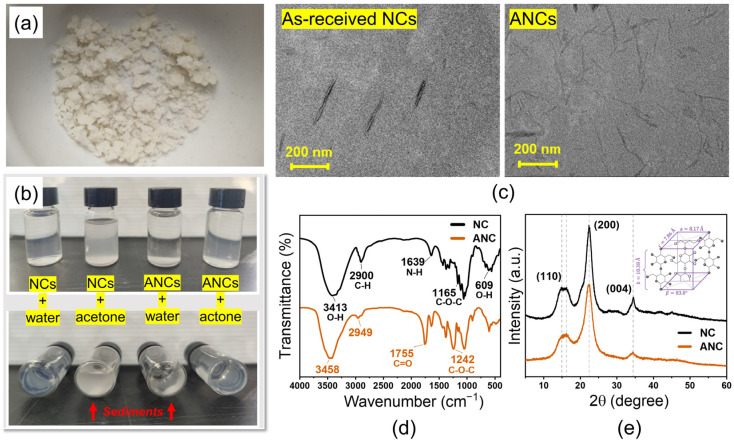
(**a**) Dried acetylated nanocelluloses (ANCs) powder. (**b**) Dispersity comparison of as-received nanocelluloses (NCs) and synthesized ANCs in water and acetone solutions after 24 h stewing. (**c**) TEM photographs of NCs and ANCs. (**d**) FTIR spectra of NCs and ANCs. New bands appeared at 1755 and 1242 cm^−1^ after acetylation. (**e**) XRD curves of NCs and ANCs accompanied with crystal lattice schematic.

Figure 1a,b, respectively show the morphology of the synthesized ANCs and the dispersity of 0.5 wt.% as-received NCs and ANCs in water and acetone after 24 h stewing. The as-received NCs demonstrate better dispersity in water than in acetone. In contrast, the ANCs tend to self-aggregate and precipitate rapidly in water while exhibiting outstanding dispersity in acetone. The change in dispersity is attributed to the weakening of intramolecular and intermolecular hydrogen bonds caused by the attached hydroxyl groups. The outstanding dispersity of ANCs in acetone lays the fundamental for achieving homogeneous dispersion in the epoxy matrix.

**Figure 2:**
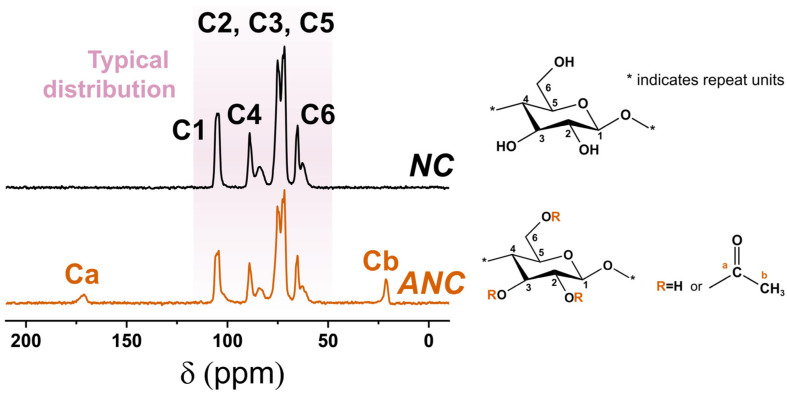
NMR spectra of as-received nanocelluloses (NCs) and acetylated nanocelluloses (ANCs), accompanied by schematic of substituted groups.

The TEM photographs of as-received NCS and ANCs are shown in Figure 1c. The needle-like as-received NCs have an average length and diameter of 318 and 25 nm, respectively. The structure of ANCs is well-preserved, while the average sizes are slightly reduced for the average length and diameter evaluated as 236 and 20 nm, respectively. In addition, the profile of ANCs becomes blurred due to the partial dissolution of cellulose molecules during acetylation. The FTIR spectra of NCs before and after acetylation are presented in Figure 1d. Wavenumbers at 3413, 2900, 1639, 1164, and 609 cm^−1^ are the characteristic peaks of NCs. The peak at 3395 cm^−1^ corresponds to the free hydroxyl groups on the surface of NCs. The spectrum of ANCs presents distinctive peaks at 1754 and 1241 cm^−1^, attributing to the C=O and C−O−C stretching of the ester bond, respectively [34]. It is noteworthy that the peak intensity at 3413 and 3457 cm^−1^, which corresponds to the hydroxyl groups, is significantly lowered after acetylation, indicating the successful substitution of acetyl groups. The XRD curves are shown in Figure 1e. The as-received NCs present a typical XRD curve of lattice type I, in which the absorption peaks mainly appear at 15° and 22.5° (2θ) with crystallinity of 76.8% [35]. Based on the Meyer–Misch model, the lattice type I is monoclinic with cell dimensions a=8.17 Å, b=10.38 Å (chain axis), c=7.86 Å, β=83.0°, and two cellobiose moieties per unit cell with densities of 1.582 and 1599 g/cm^3^ [36,37]. The ANCs after acetylation demonstrate preserved lattice structure with lowered peak intensity at 15° and 22.5° (2θ) with reduced crystallinity of 66.7%. During acetylation, the majority of hydroxyl groups are substituted by acetyl groups, and the intramolecular and intermolecular hydrogen bonds are thereby eliminated. In addition, the introduction of acetyl groups increases the steric hindrance and promotes the transformation of the crystalline region into the amorphous region. The as-received NCs exhibit typical carbon atom distribution in the NMR spectra, as shown in Figure 2. In addition to maintaining the carbon atom distribution of NCs, ANCs appear at two new peaks at 172 and 20 ppm, respectively, corresponding to −C=O and −CH_3_ in acetyl groups. In accordance with the results of diverse characterizations, the successful acetylation of NCs is proofed.

### 3.2. Mechanical Properties and SME of Different Constituted SMEPs

**Figure 3:**
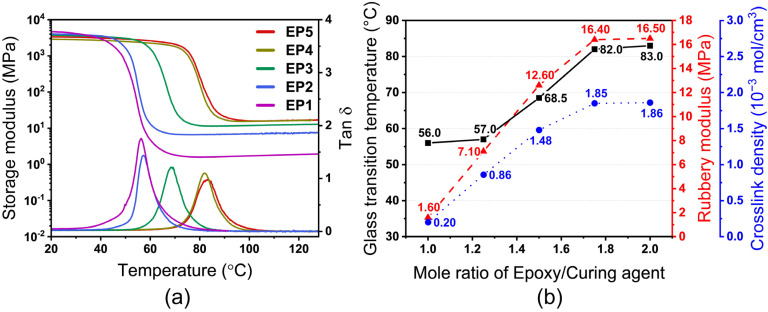
(**a**) Storage modulus and loss factor of different constituted SMEPs (EP1, EP2, EP3, EP4, EP5, respectively, correspond to mole ratio of epoxy monomer to curing agent η = 1.00, 1.25, 1.50, 1.75, 2.00). (**b**) Glass transition temperature, rubbery modulus, and crosslink density as the functions of mole ratio of epoxy monomer to curing agent.

Figure 3a shows the storage modulus and loss factor (tanδ) of different constituted SMEPs. All samples exhibit relatively constant storage modulus in both glassy ($E_{g}$) and rubbery ($E_{r}$) regions, and the storage modulus in each state presents substantial diversity of 2 to 3 orders of magnitude. According to the assumption that the polymer network has a homogeneous three-dimensional crosslink distribution, D is the crosslink density which is determined as [38]:

(1) $$D=\frac{E_{r}}{3RT}$$

where R is the gas constant, and T is the absolute temperature. The glass transition temperature of SMEPs is assigned as the temperature at the summit of the loss factor. Figure 3b presents the glass transition temperature, rubbery modulus, and crosslink density as the functions of mole ratio of epoxy to curing agent. Changing the mole ratio of epoxy to curing agent leads to different crosslink densities. The crosslink density of SMEPs presents a substantially linear relationship with epoxy/curing agent mole ratio before η=1.8, and then reaches a plateau where the crosslinking network is difficult to be further formed. As the consequence of an increased crosslink density, the mobility of macromolecular chains reduces and thus increases the glass transition temperature. Due to the positive correlation between glass transition temperature and mole ratio,$\;T_{g}$ is programmable in the range of 56 to 83 °C by changing η from 1 to 2. Similarly, the tendency of D and $E_{r}$ also coincides with $T_{g}$ that increases rapidly when η is lower than 1.75, and keeps steady afterward. Those results coincide with the well-established scientific consensus that the glass transition temperature and modulus demonstrate linear relationships with crosslink density [39,40].

**Figure 4:**
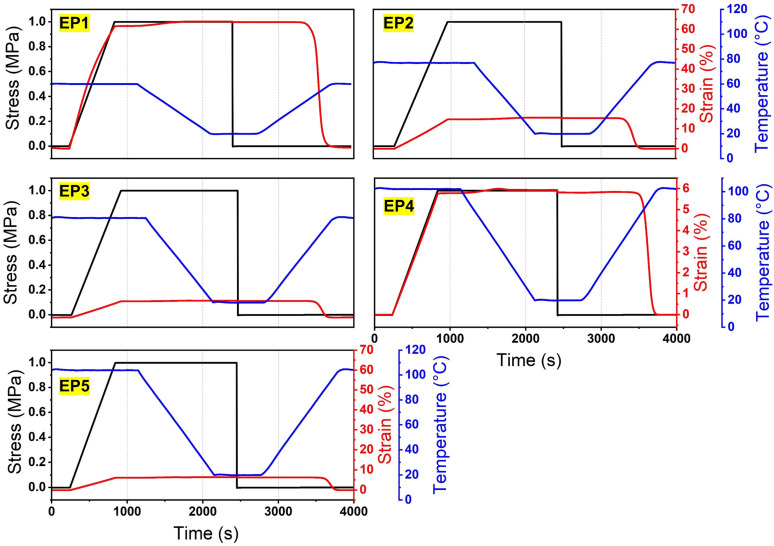
Shape memory cycle curves of different constituted SMEPs obtained by single cantilever DMA method (EP1, EP2, EP3, EP4, EP5, respectively correspond to mole ratio of epoxy monomer to curing agent η = 1.00, 1.25, 1.50, 1.75, 2.00).

**Figure 5:**
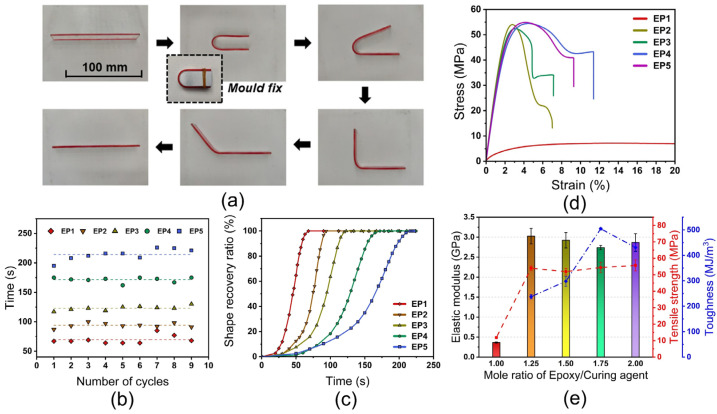
(**a**) Fold-unfold process during deployment test. (**b**) Recovery time during 9 times fold-unfold cycles (EP1, EP2, EP3, EP4, EP5, respectively, correspond to mole ratio of epoxy monomer to curing agent η = 1.00, 1.25, 1.50, 1.75, 2.00). (**c**) Recovery ratio of SMEPs as the function of time. (**d**) Representative stress–strain curves of different constituted SMEPs (**e**) Elastic modulus, tensile strength, and fracture toughness of SMEPs.

The shape memory cycle curves obtained by single cantilever DMA method are shown in Figure 4. The shape-fixity ratio $R_{f}$ and recovery ratio $R_{r}$ can be, respectively, calculated by [41]:

(2) $$R_{f}=\frac{\varepsilon_{fixed}}{\varepsilon_{max}}\times 100\%$$

(3) $$R_{r}=\frac{\varepsilon_{fixed}-\varepsilon_{final}}{\varepsilon_{fixed}}\times 100\%$$

where $\varepsilon_{fixed}$ and $\varepsilon_{final}$ are the fixed strain and residual strain after recovery, respectively. The detailed information of $T_{g}$, $E_{r}$, D, $R_{f}$, and $R_{r}$ is tabulated in Table 3. The shape-fixity and recovery ratios of all samples exceed 98.0% and 99.0%, respectively, indicating excellent SME of the fabricated SMEPs. The satisfactory SME is also validated by the deployment test, wherein the fix and recovery ratios of all samples also, respectively, exceed 98.0% and 99.0% after 9 times of the fold-unfold process, as shown in Figure 5a. Figure 5b shows the recovery time during 9 times of the fold-unfold process. The recovery time is relatively steady during multiple fold-unfold cycles, indicating good repeatability. The average recovery time of EP1 to EP5 is determined as 68, 94, 123, 172, and 216 s. With the increase of mole ratio of epoxy monomer to curing agent, the recovery time is significantly prolonged. The increased crosslink density exacerbates the intermolecular entanglements and impedes the molecular segment movements, resulting in prolonged recovery time. The relationships between the recovery ratio and recovery time of different constituted SMEPs are illustrated in Figure 5c. A three-stage shape recovery behavior is observed for all SMEP samples, wherein the shape recovery shows a higher recovery rate in the intermediate stage and a lower recovery rate in the initial and final stages. At the initial stage, it takes time for the samples to reach the recovery temperature from 10 °C, contributing to a lower recovery rate. At the intermediate stage, the mobility of molecular chains is activated by temperature stimuli, the frozen strain is released, and the shape of samples recovers rapidly under the recovery stress. At the final stage, as the frozen strain has been almost thoroughgoing consumed, the residual recovery stress becomes deficient in sustaining a high recovery rate.

Figure 5d shows the representative tensional stress–strain curves of different constituted SMEPs at room temperature. EP1 presents significantly lower elastic modulus, ultimate strength, and higher fracture strain compared to other samples. The partial intermolecular crosslinking of EP1 retains the liquidity characteristic of epoxy, and the high viscosity of EP1 results in the deterioration of the stiffness and strength, rendering it inappropriate for practical applications. EP2 to EP5 demonstrate comparative elastic modulus and ultimate strength, wherein the highest elastic modulus appears at EP2, and the highest ultimate strength is observed at EP5, as shown in Figure 5e. Although the modulus and strength of EP4 are not superlative, the elongation at break and stress at the fatal failure of EP4 are exceptional compared to other experimental groups, contributing to a significant improvement in fracture toughness of 541.3 MJ/m^3^. It is noteworthy that the fracture toughness of EP1 is 3250 MJ/m^3^ as the consequence of its superhigh fracture strain. Established on the remarkable stiffness and strength, superlative fracture toughness, EP4 with η equals to 1.75 is selected as the matrix for fabricating ANCs incorporated SMEPNs.

### 3.3. Mechanical Properties and SME of ANCs Incorporated SMEPNs

**Figure 6:**
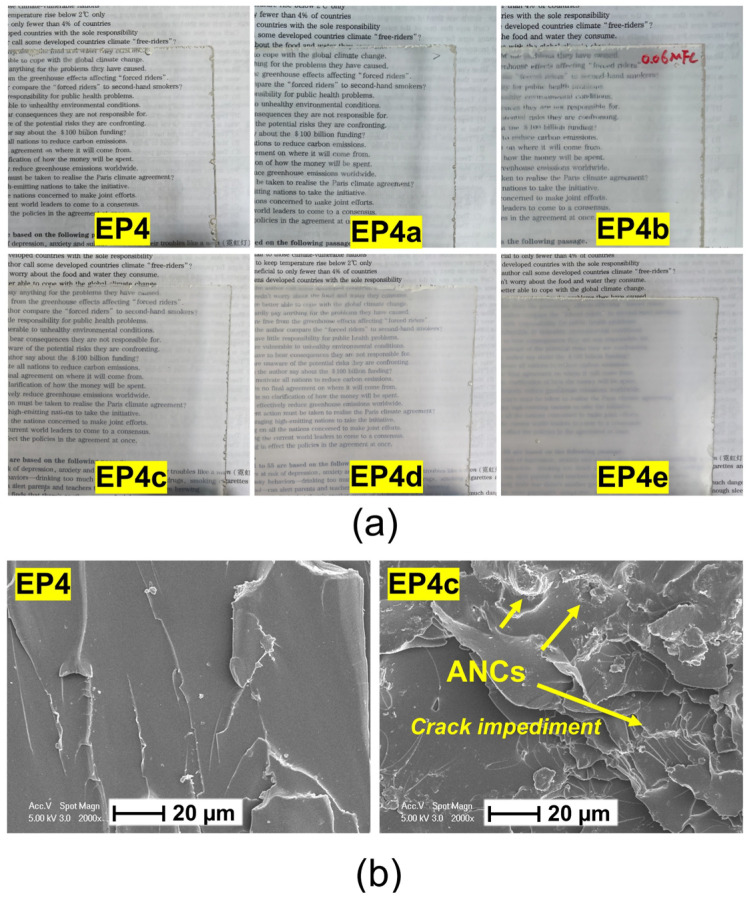
(**a**) Transparency of SMEPNs with different ANCs loading (EP4, EP4a, EP4b, EP4c, EP4d, EP4e, respectively, correspond to 0.00, 0.02, 0.06, 0.20, 0.40, 0.60 wt.% ANCs incorporated in a matrix with mole ratio of epoxy monomer to curing agent η = 1.75). (**b**) SEM photographs of fracture surfaces of EP4 and EP4c.

**Figure 7:**
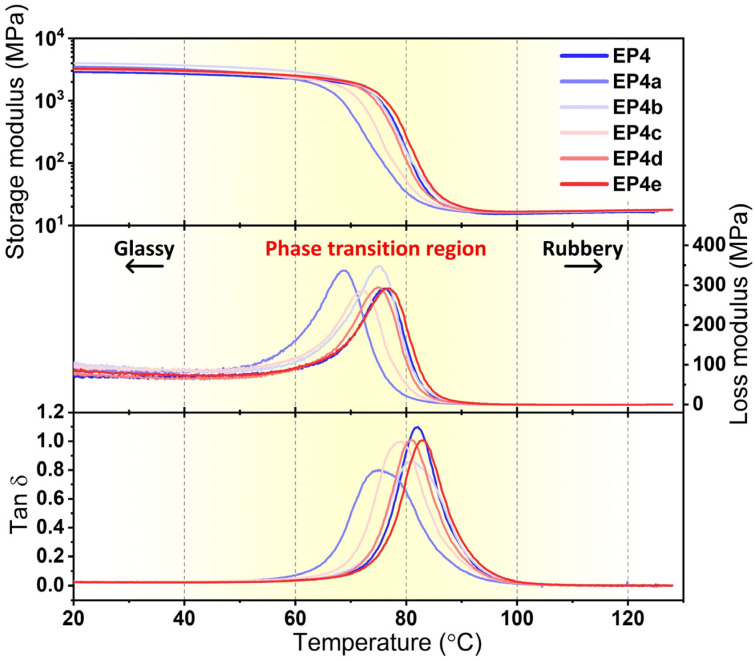
Storage modulus, loss modulus, and loss factor of ANCs incorporated SMEPNs (EP4, EP4a, EP4b, EP4c, EP4d, EP4e, respectively, correspond to 0.00, 0.02, 0.06, 0.20, 0.40, 0.60 wt.% ANCs incorporated in matrix with mole ratio of epoxy monomer to curing agent η = 1.75).

SMEPNs with epoxy monomer to curing agent mole ratio of 1.75 and ANCs loading of 0, 0.02, 0.06, 0.2, 0.4, 0.6 wt.% are, respectively abbreviated as EP4, EP4a, EP4b, EP4c, EP4d, and EP4e, as listed in Table 2. The transparency of SMEPNs reduces with the increase of ANCs loading, as shown in Figure 6a. Figure 6b presents the SEM photographs taken on the fracture surfaces of EP4 and EP4c. The pristine SMEPs without ACNs incorporation demonstrate a smooth fracture surface, indicating brittle fracture. In contrast, EP4c exhibits a rough fracture surface with multiple curved river-like patterns, revealing the transition from brittle fracture to ductile fracture. The cracks are impeded or curved by incorporated ANCs, and the crack propagation length is significantly prolonged, resulting in a larger energy requirement to rupture the specimen, and eventually leading to an enhanced toughness. Moreover, no agglomeration and pit holes are observed. The ANCs are evenly distributed with good interfacial adhesion with epoxy. The DMA results of ANCs incorporated SMEPNs are shown in Figure 7, and the detailed properties derived from DMA results are listed in Table 4. Incorporating ANCs into SMEPs remarkably increases the storage modulus at room temperature, which is attributed to the inherently high stiffness of ANCs. In the glassy region beneath about 60 °C, relevantly high storage modulus is sustained due to the lack of mobility of macromolecular chains, or so-called the frozen phases. In the glassy/rubbery transition region between about 60 °C to 85 °C, the storage modulus drastically reduces on account of the Brownian movement of macromolecular chains near $T_{g}$. With further increase in temperature, the rubbery region is eventually reached. In the rubbery region, the frozen phases completely transfer to the active phases, and the strict alignment of macromolecular chains is lost ascribed to the elevated temperature, resulting in 2 to 3 orders of magnitude reduction in the storage modulus.

The loss modulus is considered as the viscous response of SMEPNs, which describes the phenomenon that the energy of the material is consumed in the form of thermal energy during deformation. Incorporating 0.06% ANCs contributes to the highest peak value of the loss modulus. A higher loss modulus indicates the evener distribution of nano-additives. While agglomeration or uneven distribution leads to a two-phase heterogeneous material system rather than a homogeneous composite, which eventually results in the reduction of energy consumption during dynamic loading [42,43]. The loss modulus of all samples drops to near zero when the temperature is above $T_{g}$. In the rubber region, the macromolecular chains possess very high mobility, and the shear friction between ANCs and macromolecular chains becomes negligible. The loss factor is determined as the proportion between the storage modulus and the loss modulus, indicating the damping property of materials. EP4a and EP4b possess lower loss factors, which reveals the effective impediment of macromolecular chain mobility by evenly distributed ANCs. Moreover, the peak width of the loss factor is increased after ANCs incorporation, which is attributed to the reduction of chain mobility and the increase of relaxing time, implying the fine interfacial adhesion between ANCs and epoxy matrix. The $T_{g}$ of SMEPNs is evaluated by the temperature at the summit of the loss factor, as shown in Table 4. Incorporating ANCs results in the reduced $T_{g}$ except for EP4e, which coincides with the research of Liu et al. [44]. At lower concentrations, the good miscibility and the interpenetrating nanocrystals contribute to the weakened interactions between epoxy chains, which eventually results in the lowered $T_{g}$ values [45]. Nevertheless, excessive ACNs loading in EP4e leads to agglomerations and thus severely impedes chain migration, contributing to the increase of $T_{g}$.

**Figure 8:**
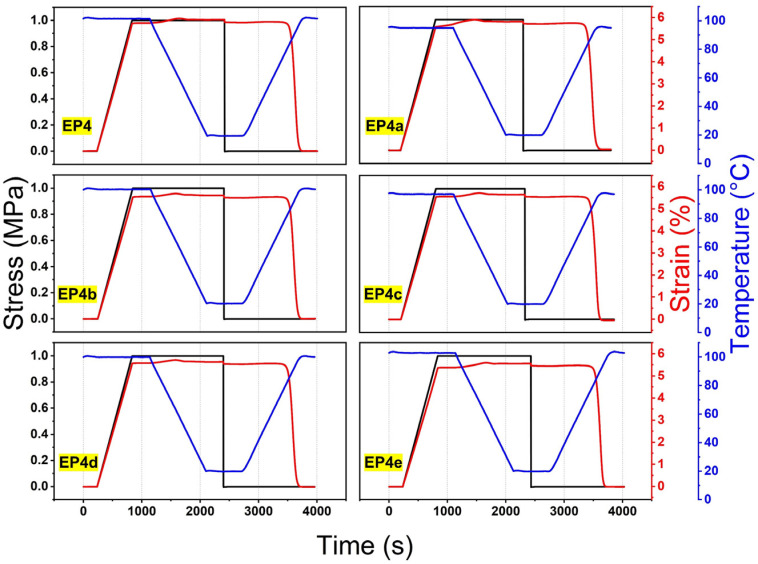
Shape memory cycle curves of SMEPNs with different ANCs loading amounts (EP4, EP4a, EP4b, EP4c, EP4d, EP4e, respectively, correspond to 0.00, 0.02, 0.06, 0.20, 0.40, 0.60 wt.% ANCs incorporated in matrix with mole ratio of epoxy monomer to curing agent η = 1.75).

**Figure 9:**
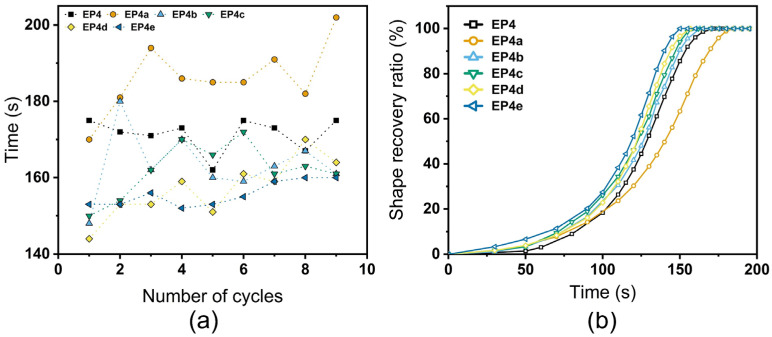
(**a**) Average recovery time during 9 times fold-unfold process. (**b**) Recovery ratio as the function of deployment time of ANCs incorporated SMEPNs (EP4, EP4a, EP4b, EP4c, EP4d, EP4e, respectively, correspond to 0.00, 0.02, 0.06, 0.20, 0.40, 0.60 wt.% ANCs incorporated in matrix with mole ratio of epoxy monomer to curing agent η = 1.75).

The shape memory cycle curves of SMEPNs obtained by single cantilever DMA method are illustrated in Figure 8. The SME is completely preserved after ANCs incorporation, of which the fix and recovery ratio of all samples, respectively, exceed 98.0% and 99.0%, as shown in Table 4. The average recovery time during the 9 times fold-unfold process and the shape recovery ratio as the function of deployment time are illustrated in Figure 9. The recovery time is evaluated as 172, 186, 163, 162, 157, and 155 s for EP4 to EP4e, respectively. Comprehensively, the shape recovery accelerates with higher loading of ANCs, while the stability of the recovery time during multiple deployments slightly deteriorates after ANCs incorporation.

**Figure 10:**
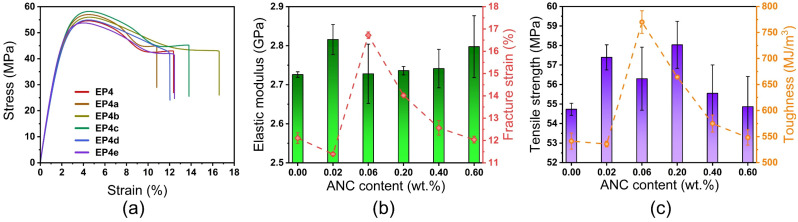
(**a**) Representative strain-stress curves of ANCs incorporated SMEPNs (EP4, EP4a, EP4b, EP4c, EP4d, EP4e, respectively, correspond to 0.00, 0.02, 0.06, 0.20, 0.40, 0.60 wt.% ANCs incorporated in matrix with mole ratio of epoxy monomer to curing agent η = 1.75). (**b**) Elastic modulus and fracture strain of SMEPNs with different ANCs loading. (**c**) Tensile strength and toughness of SMEPNs with different ANCs loading.

The representative stress–strain curves indicate that all samples perform yield behavior and plastic deformation during tensile test, as shown in Figure 10a. The elastic modulus, tensile strength, fracture strain, and fracture toughness of ANCs incorporated SMEPNs are presented in Figure 10b,c. The incorporation of ANCs moderately improves the elastic modulus and the ultimate strength. For instance, EP4a increases the elastic modulus by 3.3%, and EP4c increases the ultimate strength by 6.0%. Due to the low loading of ANCs, the stiffening and strengthening effects are beneath significant. The tendency of the fracture strain and fracture toughness regarding ANCs loading is identical that increases first and then decreases. Compared to EP4, EP4e perform the most remarkable improvement on the fracture strain and the toughness for 15.8% and 22.7% without compromise of the elastic modulus. The extremely low loading of ANCs in EP4a plays a role of the defects rather than the reinforcements, resulting in the deterioration of the fracture strain and the toughness. The fracture strain and the toughness are also lowered for samples having high ANCs loadings like EP4d and EP4e. The excessively incorporated ANCs tend to be agglomerated in matrix and thereby reduce the effective loading transfer between ANCs and epoxy matrix, resulting in the deterioration of ductility.

## 4. Conclusions

SMEPs with different crosslink densities are successfully prepared by adjusting the mole ratio of epoxy monomer to curing agent. The different constituted SMEPs demonstrate relatively constant and discrepant storage modulus in their respective glassy and rubbery regions, which confirms the typical SMP characteristics. Each constitution exhibits a remarkable shape-fixity ratio and recovery ratio, and the SME keeps its consistency well during multiple deployments. Glass transition temperature and recovery time are two programmable parameters because they show positive correlations regarding the crosslink density. The as-received NCs are successfully acetylated, which is validated by the appearance of bands at 1754, 1380, and 1241 cm^−1^ in FTIR spectra, and the new carbon atom distributions at 172 and 20 ppm in NMR spectra. The prepared ANCs demonstrate outstanding dispersity in acetone solution which is attributed to the weakening of intramolecular and intermolecular hydrogen bonds caused by the attached hydroxyl groups. ANCs incorporated SMEPNs are successfully synthesized with a matrix constituted with 1.75 mole ratio of epoxy monomer to curing agent. The transparency of the SMEPNs decreased with the increasing loading of ANCs. The incorporation of ANCs improves the storage modulus without compromising on glass transition temperature. The shape memory effect of the synthesized SMEPNs is sustained, of which the shape-fixity ratio and the recovery ratio exceed 98% and 99%, respectively. The recovery time is comprehensively reduced with higher ANCs loading. On the mechanical property aspect, the optimal ANCs loading is preferred to enhance the elastic modulus, tensile strength, fracture strain, and toughness. The homogeneously dispersed ANCs play a crucial role in pinning the cracks and prolonging the crack propagation length, contributing to the exceptional enhancement of the fracture strain and toughness. This study is expected to provide valuable inspiration in exploring the nature-derived NCs and their nanocomposites, and to expand the scope of their intelligent applications in textiles, flexible electronics, and metamaterials.

## Figures and Tables

**Table 1:** Detailed composition of prepared SMEPs.

Abbreviation	EP1	EP2	EP3	EP4	EP5
Mole ratio of D230 to EPON 826 (η)	1.00	1.25	1.50	1.75	2.00
EPON 826 (g)	50	50	50	50	50
D230 (g)	31.58	25.27	21.05	18.05	15.79

**Table 2:** Detailed composition of prepared SMEPNs.

Abbreviation	ANCs (wt.%)	ANCs (g)	EPON826 (g)	D230 (g)
EP4	0.00	0.00	50	18.05
EP4a	0.02	0.01	50	18.05
EP4b	0.06	0.03	50	18.05
EP4c	0.20	0.10	50	18.05
EP4d	0.40	0.20	50	18.05
EP4e	0.60	0.30	50	18.05

**Table 3:** Detailed information of fabricated different constituted SMEPs.

	$T_{g}$ (°C)	$E_{r}$ (MPa)	D (10^−3^ mol/cm^3^)	$R_{f}$ (%)	$R_{r}$ (%)
EP1	56	1.6	0.20	99.6	99.2
EP2	57	7.1	0.86	99.0	100
EP3	68.5	12.6	1.48	98.6	99.8
EP4	82	16.4	1.85	98.0	99.9
EP5	83	16.5	1.86	98.0	100

**Table 4:** Dynamic mechanical properties and recovery ability of SMEPNs.

	$T_{g}$ (°C)	$E_{g}$ (MPa)	$E_{r}$ (MPa)	$R_{f}$ (%)	$R_{r}$ (%)
EP4	82.1	2883.6	15.3	98.0	99.9
EP4a	75.0	3504.3	16.0	98.3	99.3
EP4b	80.8	3998.5	16.0	98.2	99.9
EP4c	78.8	3125.1	16.3	98.3	100.0
EP4d	80.8	3208.2	16.3	98.2	100.0
EP4e	82.8	3268.0	16.5	98.0	100.0

## Data Availability

The data presented in this study are available on request from the corresponding author. The data are not publicly available due to privacy.
